# Supplementary material for: Molecular Epidemiology and Evolutionary Dynamics of Human Influenza Type-A Viruses in Africa: A Systematic Review
Source: Microorganisms. 2022 Apr 25;10(5):900. doi: 10.3390/microorganisms10050900 (PMC9145646; doi:10.3390/microorganisms10050900)
Supplement: Supplementary file 1 [file microorganisms-10-00900-s001.zip › microorganisms-1619586-supplementary.pdf]

TABLE OF CONTENTS

Table S1: Terms and definitions 2

Supplementary Methods *4*

Table S2: Keywords and their corresponding mesh terms included in the search strategy 4

Detailed Search Strategy for PubMed 5

Table S3: Study article inclusion and exclusion criteria 6

Table S4: Study quality and risk-of-bias assessment scheme 7

Supplementary Results *9*

Table S5: List of all African countries with or without viral sequence data analysed in the included studies 9

Table S6: Study quality and risk of bias assessment 10

Table S7: Detailed study sampling bias assessment 14

Table S8: Circulating genetic clades among Africa H1N1 viruses sampled between 2001 and 2009 23

Table S9: Circulating genetic clades among Africa H1N1pdm09 viruses between 2009 and 2018 ... 24

Figure S10: Viral diversification and distribution of genetic clades among H1N1pdm09 viruses that circulated in Africa versus elsewhere during the 2009-2020 seasons. 26

Table S11: Circulating genetic clades among Africa H3N2 viruses between 2004 and 2018 27

Figure S12: Viral diversification and distribution of genetic clades among H3N2 viruses that circulated in Africa versus elsewhere during the 2009-2020 seasons. 30

Table S1: Terms and definitions

Term	Definition
**Influenza virus**	Virus belonging to Orthomyxoviridae family which causes the influenza infection
**Basic reproductive number (R_0)**	Probability of transmission of a pathogen like a virus in absence of an intervention or number of secondary infections caused by a primary infection in absence of an intervention.
**Subtype**	Viruses with a specific combination of hemagglutinin (HA) and Neuraminidase (NA) genes
**Deoxyribonucleic acid (DNA)**	Sequence of nucleotides present within organisms
**Mutation**	Change in the composition of nucleotides or amino acid sequence. This could be a deletion, substitution, or insertion
**Mutation or substitution rate**	The rate at which amino acids at a given site change or get substituted per a given time (especially per year)
**Drug resistance**	The loss of susceptibility or response to a drug. In this case, the resistant influenza viruses carry NA and Matrix protein genes with specific amino acid that make them non-reactive to drugs.
**Cluster**	In reference to molecular epidemiology, a cluster is a group of viruses with a similar epidemiological or genetic history
**Clade or genetic clade**	In reference to molecular epidemiology, a genetic clade is group of viruses with a similar amino acid substitution in their HA1 or HA2 proteins
**Vaccine efficacy**	Is the degree at which the disease attack rate reduces after vaccination. An attack rate is the proportion of an exposed population that gets infected with a disease during a specified time interval.
**$p_{epitope}$ model**	A statistical tool used to predict vaccine efficacy between vaccines and circulating viruses based on amino acids in the dominant antigenic sites or epitopes in the hemagglutinin (HA) proteins the model measures of antigenic distance ($p_{epitope}$) between the vaccine and circulating viruses and makes prediction of vaccine efficacy. $p_{epitope}$ = number of amino acids substitutions in dominant epitope divide by total number of amino acids in dominant epitope. The higher the $p_{epitope}$, the lower the vaccine efficacy(Bonomo and Deem, 2018)
**Phylodynamics**	Study of evolutionary processes that shape the phylogenies such as changes in viral effective population sizes [Ne(t)], substitution rates (amino acid substitutions per site per time), selection pressures, probability of transmission (basic reproduction ratio, R_0)), and reassortment
**Phylogeography**	Study of geographical distribution or migration patterns of given organisms for example viruses
**Reassortment**	Exchange of gene segments between viruses co-infecting a cell during viral replication.
**Gene**	A distinct sequence of nucleotides that code for a protein
**Genome**	All of the genetic material within an organism
**Isolate**	A culture of a microorganism
**Hemagglutination inhibition (HAI) assay**	Hemagglutination inhibition (HAI) assay is a method used to measure the antigenic variation or detect and quantify functional antibodies in a patient's serum against influenza infection. The assay is based on the ability of the viral surface proteins (HA) to bind to sialic acids on red blood cells (RBCs) forming an agglutinate or clump. In the presence of antibodies (Abs) in the serum, the agglutination is blocked and the antibody concentrations are reported as titres. The higher the titre values the higher the antibody concentrations. The titre value correlates with the affinity of an antiserum (Abs) to a given virus; thus, the HAI assay has widely been used to determine antigenic ability of circulating viruses. The HAI assay complements sequence analysis as it factors in structural and antigenic elements of the HA gene absent when comparing sequence data alone(Hirst, 1943).
**Molecular epidemiology**	The use of molecular typing methods for infectious agents in the study of the distribution, dynamics, and determinants of health and disease in human populations
**Neuraminidase inhibition (NAI) or enzyme inhibition assay**	Neuraminidase inhibition (NAI) or enzyme inhibition assay is a method used to determine the activity or ability of neuraminidase protein to elution influenza viruses bound to the red blood cells as well as test the sensitivity of circulating viruses to Neuraminidase inhibition (NAI) dugs. One common assay is the use of a fluorescent-based substrate 2 ′ -(4-methylumbelliferyl)- α -D- N -acetylneuraminic acid (MUNANA). In presence of Neuraminidase, the MUNANA substrate is cleaved detected as florescence. The amount of fluorescence is directly proportional to the enzyme (NA) activity. The NAI assay results are reported as IC_{50} which is the drug concentration required for 50% inhibition of enzyme(Aymard-Henry et al., 1973)
**Nucleotide**	The basic structure of nucleic acids, such as DNA
**Pyrosequencing**	Pyrosequencing is a real-time PCR-based sequencing method used for rapid detection of and quantification of markers of drug resistance. It is based on the pyrophosphate (PPi) released during the DNA polymerase reaction, which is converted to ATP and quantified and produces light. Best for known markers.
**A time-resolved phylogeny**	A time-resolved phylogeny (TRP) is a tree whose branch lengths are proportional to the time since the most recent common ancestor (TMRCA), and nodes correspond to the inferred dates of extinct or sampled viruses (Volz et al., 2013; Sagulenko et al., 2018).

**HYPHY**	Hypothesis testing using phylogenies (http://hyphy.org/)
**Fixed effects likelihood (FEL)**	Maximum likelihood method used for identifying sites that may have experienced pervasive diversifying or purifying selection
**Single likelihood ancestor counting (SLAC)**	Substitution counting-based method for identifying sites that may have experienced pervasive diversifying or purifying selection.
**Surveillance**	In the context of healthcare, surveillance is the collection, analysis and interpretation of health-related data used to monitor the trends and design interventions for public health
**Whole genome sequencing (WGS)**	The process of identifying all the nucleotides in the DNA of an organism

Supplementary Methods

Table S2: Keywords and their corresponding mesh terms included in the search strategy

Keyword or Concept	Keyword group or MeSH terms
**influenza**	"influenza" OR "human influenza" OR "influenzas" OR "Flu" OR "pandemic influenza" OR "pandemic flu" OR "seasonal influenza" OR "seasonal flu" OR "influenza virus*"
**Sequencing, genomic and phylogenetic analysis**	"Amino acid sequence analysis" OR "Emergence" OR "Evolution" OR "Evolutionary Dynamics" OR "Genetic analysis" OR "Genome analysis" OR "Genetic characteri*" OR "Genetic diversity" OR "Genetic sequencing" OR "Molecular evolution" OR "Molecular characteri*" OR "Molecular analysis" OR "Molecular epidemiology" OR "Molecular Surveillance" OR "Phylogenetic analysis" OR "Phylogenetic characteri*" OR "Phylodynamics" OR "Phylogeography" OR "Phylogeographical analysis" OR "Sequence analysis" OR "Whole-genome analysis" OR "Whole genome characteri*" OR "Whole genome sequencing"
**Location**	"Algeria" OR "Angola" OR "Atlantic Islands" OR "Benin" OR "Botswana" OR "Burkina Faso" OR "Burundi" OR "Cameroon" OR "Cape Verde" OR "Central African Republic" OR "Chad" OR "Comoros" OR "Congo" OR "Democratic Republic of the Congo" OR "DRC" OR "Côte d'Ivoire" OR "Ivory Coast" OR "Djibouti" OR "Egypt" OR "Equatorial Guinea" OR "Eritrea" OR "Eswatini" OR "Ethiopia" OR "Gabon" OR "Gambia" OR "Ghana" OR "Guinea" OR "Guinea-Bissau" OR "Ivory Coast "OR "Kenya" OR "Lesotho" OR "Liberia" OR "Libya" OR "Madagascar" OR "Malawi" OR "Mali" OR "Mauritania" OR "Mauritius" OR "Mayotte" OR "Morocco" OR "Mozambique" OR "Namibia" OR "Niger" OR "Nigeria" OR "Republic of the Congo" OR "Réunion" OR "Reunion" OR "Rwanda" OR "Sahrawi Arab Democratic Republic" OR "Saint Helena" OR "São Tomé and Príncipe" OR "Sao Tome and Principe" OR "Senegal" OR "Seychelles" OR "Sierra Leone" OR "Somalia" OR "Somaliland" OR "South Africa" OR "South Sudan" OR "Sudan" OR "Swaziland" OR "Tanzania" OR "Togo" OR "Tunisia" OR "Uganda" OR "Western Sahara" OR "Zambia" OR "Zimbabwe" OR "Central Africa" OR "East Africa" OR "Eastern Africa" OR "North Africa" OR "Northern Africa" OR "Southern Africa" OR "West Africa" OR "Western Africa" OR "Africa" OR "Sub-Saharan" OR "Sub Saharan" OR "African Continental Ancestry Group"
**Exclusion**	"Avian" OR "Avian influenza" OR "Avian flu" OR "Bird flu" OR "Covid-19" OR "Coronavirus" OR "Corona virus" OR "Chikungunya" OR "Crimean-Congo haemorrhagic fever" OR "CHF"OR "Dengue" OR "Ebola" OR "Ebola virus*" OR "equine" OR "Guinea Pig*" OR "HIV1" OR "HIV-1" OR "H5N1" OR "H5N2" OR "H5N5" OR "H5N6" OR "H5N8" OR "H7N9" OR "H9N2" OR "H. influenza" OR "Haemophilus" OR "mycoplasma pneumonia*" OR "pig*" OR "pneumonia*" OR "rhinovirus*" OR "respiratory syncytial virus" OR "RSV" OR "swine" OR "swine flu" OR "zika" OR "zika virus*"

Detailed Search Strategy for PubMed

(((("influenza"[Title/Abstract] OR "human influenza"[Title/Abstract] OR "influenzas"[Title/Abstract] OR "Flu"[Title/Abstract] OR "pandemic influenza"[Title/Abstract] OR "pandemic flu"[Title/Abstract] OR "seasonal influenza"[Title/Abstract] OR "seasonal flu"[Title/Abstract] OR "influenza virus*")[Title/Abstract]) AND (("Amino acid sequence analysis"[Title/Abstract] OR "Emergence"[Title/Abstract] OR "Evolution"[Title/Abstract] OR "Evolutionary Dynamics"[Title/Abstract] OR "Genetic analysis"[Title/Abstract] OR "Genome analysis"[Title/Abstract] OR "Genetic characteri*"[Title/Abstract] OR "Genetic diversity"[Title/Abstract] OR "Genetic sequencing"[Title/Abstract] OR "Molecular evolution"[Title/Abstract] OR "Molecular characteri*"[Title/Abstract] OR "Molecular analysis"[Title/Abstract] OR "Molecular epidemiology"[Title/Abstract] OR "Molecular Surveillance"[Title/Abstract] OR "Phylogenetic analysis"[Title/Abstract] OR "Phylogenetic characteri*"[Title/Abstract] OR "Phylodynamics"[Title/Abstract] OR "Phylogeography"[Title/Abstract] OR "Phylogeographical analysis"[Title/Abstract] OR "Sequence analysis"[Title/Abstract] OR "Whole-genome analysis"[Title/Abstract] OR "Whole genome characteri*"[Title/Abstract] OR "Whole genome sequencing")[Title/Abstract])) AND ("Algeria"[Title/Abstract] OR "Angola"[Title/Abstract] OR "Atlantic Islands"[Title/Abstract] OR "Benin"[Title/Abstract] OR "Botswana"[Title/Abstract] OR "Burkina Faso"[Title/Abstract] OR "Burundi"[Title/Abstract] OR "Cameroon"[Title/Abstract] OR "Cape Verde"[Title/Abstract] OR "Central African Republic"[Title/Abstract] OR "Chad"[Title/Abstract] OR "Comoros"[Title/Abstract] OR "Congo"[Title/Abstract] OR "Democratic Republic of the Congo"[Title/Abstract] OR "DRC"[Title/Abstract] OR "Côte d'Ivoire"[Title/Abstract] OR "Ivory Coast"[Title/Abstract] OR "Djibouti"[Title/Abstract] OR "Egypt"[Title/Abstract] OR "Equatorial Guinea"[Title/Abstract] OR "Eritrea"[Title/Abstract] OR "Eswatini"[Title/Abstract] OR "Ethiopia"[Title/Abstract] OR "Gabon"[Title/Abstract] OR "Gambia"[Title/Abstract] OR "Ghana"[Title/Abstract] OR "Guinea"[Title/Abstract] OR "Guinea-Bissau"[Title/Abstract] OR "Ivory Coast "OR "Kenya"[Title/Abstract] OR "Lesotho"[Title/Abstract] OR "Liberia"[Title/Abstract] OR "Libya"[Title/Abstract] OR "Madagascar"[Title/Abstract] OR "Malawi"[Title/Abstract] OR "Mali"[Title/Abstract] OR "Mauritania"[Title/Abstract] OR "Mauritius"[Title/Abstract] OR "Mayotte"[Title/Abstract] OR "Morocco"[Title/Abstract] OR "Mozambique"[Title/Abstract] OR "Namibia"[Title/Abstract] OR "Niger"[Title/Abstract] OR "Nigeria"[Title/Abstract] OR "Republic of the Congo"[Title/Abstract] OR "Réunion"[Title/Abstract] OR "Reunion"[Title/Abstract] OR "Rwanda"[Title/Abstract] OR "Sahrawi Arab Democratic Republic"[Title/Abstract] OR "Saint Helena"[Title/Abstract] OR "São Tomé"[Title/Abstract] OR "Príncipe"[Title/Abstract] OR "Sao Tome"[Title/Abstract] OR "Principe"[Title/Abstract] OR "Senegal"[Title/Abstract] OR "Seychelles"[Title/Abstract] OR "Sierra Leone"[Title/Abstract] OR "Somalia"[Title/Abstract] OR "Somaliland"[Title/Abstract] OR "South Africa"[Title/Abstract] OR "South Sudan"[Title/Abstract] OR "Sudan"[Title/Abstract] OR "Swaziland"[Title/Abstract] OR "Tanzania"[Title/Abstract] OR "Togo"[Title/Abstract] OR "Tunisia"[Title/Abstract] OR "Uganda"[Title/Abstract] OR "Western Sahara"[Title/Abstract] OR "Zambia"[Title/Abstract] OR "Zimbabwe"[Title/Abstract] OR "Central Africa"[Title/Abstract] OR "East Africa"[Title/Abstract] OR "Eastern Africa"[Title/Abstract] OR "North Africa"[Title/Abstract] OR "Northern Africa"[Title/Abstract] OR "Southern Africa"[Title/Abstract] OR "West Africa"[Title/Abstract] OR "Western Africa"[Title/Abstract] OR "Africa"[Title/Abstract] OR "Sub-Saharan"[Title/Abstract] OR "Sub Saharan"[Title/Abstract] OR "African Continental Ancestry Group"[Title/Abstract])) NOT ("Avian"[Title/Abstract] OR "Avian influenza"[Title/Abstract] OR "Avian flu"[Title/Abstract] OR "Bird flu"[Title/Abstract] OR "Covid-19"[Title/Abstract] OR "Coronavirus"[Title/Abstract] OR "Corona virus"[Title/Abstract] OR "Chikungunya"[Title/Abstract] OR "Crimean-Congo haemorrhagic fever"[Title/Abstract] OR "CHF"OR "Dengue"[Title/Abstract] OR "Ebola"[Title/Abstract] OR "Ebola virus*"[Title/Abstract] OR "equine"[Title/Abstract] OR "Guinea Pig*"[Title/Abstract] OR "HIV1"[Title/Abstract] OR "HIV-1"[Title/Abstract] OR "H5N1"[Title/Abstract] OR "H5N2"[Title/Abstract] OR "H5N5"[Title/Abstract] OR "H5N6"[Title/Abstract] OR "H5N8"[Title/Abstract] OR "H7N9"[Title/Abstract] OR "H9N2"[Title/Abstract] OR "H. influenza"[Title/Abstract] OR "Haemophilus"[Title/Abstract] OR "mycoplasma pneumonia*"[Title/Abstract] OR "pig*"[Title/Abstract] OR "pneumonia*"[Title/Abstract] OR "rhinovirus*"[Title/Abstract] OR "respiratory syncytial virus"[Title/Abstract] OR "RSV"[Title/Abstract] OR "swine"[Title/Abstract] OR "swine flu"[Title/Abstract] OR "zika"[Title/Abstract] OR "zika virus*"[Title/Abstract])

Table S3: Study article inclusion and exclusion criteria

Study characteristic	Details
**Study type or design**	✓ Molecular epidemiology<br>✓ Original studies: These could be part of already existing surveillance programme or independent clinical or research studies that sequence or use Influenza viral genomes to study viral evolution, diversity, transmission and distribution.<br>✓ The target study should have used either cell culture-based or molecular method to detect and subtype influenza, specifically a PCR or RT-PCR, but also went ahead to characterize viral evolution, transmission or migration dynamics at a molecular level (using genomic data) using any sequencing and phylogenetic analysis tool
**Study population**	✓ The target study population should be human of African ethnicity with any age and gender. These should be living and their samples collected in an African country.
**Influenza diagnosis or confirmation**	✓ The study should have used either cell culture-based method or the polymerase chain reaction (PCR or RT-PCR) to confirm presence of influenza in samples before sequencing.
**Time period**	✓ No restrictions. The study should collect data for any period of time ranging from months to years.
**Language**	✓ No restrictions.
**Location**	✓ No restrictions, as long as includes viruses isolated from African populations. For example, global studies could use online sequences isolated from Africa in their analysis
**Exclusion**	✓ Studies that did not sequence or use genomic data to make inferences even though they confirmed influenza by PCR were be excluded.<br>✓ Studies which analyzed other types or strains of human influenza other than type-A, such as B or C, and animal strains such as swine, equine and avian influenza were be excluded.<br>✓ Studies that isolated and analyzed human influenza A in animals.<br>✓ No detailed methods for example conference abstracts and presentations.

Table S4: Study quality and risk-of-bias assessment scheme

QUALITY AND RISK OF BIAS ASSESSMENT BASED ON NEWCASTLE-OTTAWA QUALITY ASSESSMENT SCALE (FOR CROSS-SECTIONAL STUDIES) AND STROME-ID CHECKLISTS

Definitions and basis of judgement

1. SELECTION BIAS: (Maximum 15 stars)

1.1 ***Site selection:***

- **a)** Has the study sampled widely or analysed sequences from more than two African sites or regions or districts or provinces country-wide or countries? **
- **b)** Has the study sufficiently described how and why they selected and sampled given sites? *
- **c)** No description

1.2 Uniform or comparable sampling across sites

- **a)** Have the sites been uniformly sampled (comparable sample numbers across sites)? **
- **b)** Sample numbers across sites not comparable (sites not uniformly sampled)
- **c)** No description on sample numbers per site

1.3 Representativeness of the sample: (target population: Influenza infections. Did they sample every person randomly or based on criteria i.e., ILI (non-random)?

- **a)** Truly representative of the average in the target population *** (all subjects or random sampling)
- **b)** Somewhat representative of the average in the target population ** (non- random sampling)
- **c)** Selected group of target population e.g., particular age group*
- **d)** No description of the sampling strategy

1.4 ***Sample size:***

- **a)** Justified and satisfactory i.e., sample number calculation**
- **b)** > 20 (to obtain accurate phylogenies with >70% bootstraps) as described by Heath et al., 2008, but not justified and satisfactory *
- **c)** Sequenced less than 20 samples but included other Africa sequences to make 20 or more for analysis*
- **d)** Not justified (i.e., less than 20)

1.5 ***Sampling Duration:*** Enough sampling to detect sufficient genomic changes

- **a)** > 1 year***. Usually, duration of a pandemic wave
- **b)** 6-12 months**
- **c)** <6 months*

1.6 Genome length:

- ***a)*** All 8 gene segments or concatenated whole-genome analysed ***
- ***b)*** Partial genome (>1 gene segment but not 8) analysed **
- ***c)*** One or partial gene segment *

2. Comparability: (Maximum 3 stars)

2.1 The subjects in different outcome groups are comparable, based on the study design or analysis. Confounding factors are controlled.

- **a)** The study controls for more than one important factor age or gender and/or location***
- **b)** The study controls for the most important factor age *
- **c)** The study controls for any additional factor, gender *
- **d)** The study controls for any additional factor, location *

3. Attrition bias or Missing data (Maximum 3 stars)

3.1 Incomplete outcome data.

- ***a)*** Were all eligible isolates sequenced and analysed? ***
- ***b)*** Was all epidemiological or demographic and clinical data available for sequenced isolates? **
- ***c)*** Only date and location of sample collection available*
- ***d)*** Does the study describe how the amount and nature of results or data dropped during the study or exclusions from the analysis and how they handled missing data? This could be due enrolled participants were later found to be ineligible, participants withdraw, lost to follow-up or data lost *
- ***e)*** No description

4. Outcome: (Maximum 2 stars)

4.1 Assessment of the outcome:

a) **Independent or blind assessment** Independent or blind assessment stated in the paper, or confirmation of the outcome by reference to secure records (health records, etc.) **

b) Record linkage (e.g., identified through ICD codes on database records or clinical symptoms) **

c) Self-report (i.e., no reference to original health records or documented source to confirm the outcome) *

d) No description

5. Reporting bias (Maximum 1 star)

5.1 Selective reporting.

a) Did the study pre-specify their outcomes and report all? *

b) Or did the researchers selectively report their outcomes i.e., reporting just a subset of the original outcome variables? For example, a study might not report results that are not statistically significant or some of the sequenced samples?

Supplementary Results

Table S5: List of all African countries with or without viral sequence data analysed in the included studies

Country	Country	Country
1. Algeria	2. Gambia	3. Reunion
4. Angola	5. Ghana	6. Rwanda
7. Benin	8. Guinea	9. Sao Tome and Principe
10. Botswana	11. Guinea-Bissau	12. Senegal
13. Burkina Faso	14. Kenya	15. Seychelles
16. Burundi	17. Lesotho	18. Sierra Leone
19. Cameroon	20. Liberia	21. Somalia
22. Cape Verde	23. Libya	24. South Africa
25. Central African Republic (CAR)	26. Madagascar	27. South Sudan or Sudan
28. Chad	29. Malawi	30. Swaziland
31. Comoros	32. Mali	33. Tanzania
34. Ivory Coast (Ivory Coast)	35. Mauritania	36. Togo
37. Democratic Republic of Congo (DRC) or Congo	38. Mauritius	39. Tunisia
40. Djibouti	41. Mayotte	42. Uganda
43. Egypt	44. Morocco	45. Zambia
46. Equatorial Guinea	47. Mozambique	48. Zimbabwe
49. Eritrea	50. Namibia	
51. Ethiopia	52. Niger	
53. Gabon	54. Nigeria	

Table S6: Study quality and risk of bias assessment

STUDY	SELECTION BIAS						COMPARABILITY	ATTRITION BIAS	OUTCOME	REPORTING BIAS	SCORE (X/23)
	Site	Sample Comparability across sites	Representativeness of the samples	Sample size	Duration of sampling	Genome length	Controlling Confounding factors	Incomplete outcome data	Outcome assessment	Selective reporting	
**H1N1 viruses**											
Besselaar (2008)(Besselaar et al., 2008)	**		**	*	*	**		**	*	*	12
Bulimo (2012)(Bulimo et al., 2012a)	**		**	*	***	*		*	*	*	12
Hurt (2009) (Hurt et al., 2009)	**			*	***	**		*	*	*	11
Njouom (2010)(Njouom et al., 2010)	**		**		**	**		**	*	*	12
Dia (2013)(Dia et al., 2013a)			**		*	**		*	*		7
**H1N1pdm09 viruses**											
Adeola (2019)(Adeola et al., 2019)	**		**		***	*		**	*		11
Aspinall (2013)(Aspinall et al., 2013)	**		**	**	**	*		**	*	*	13
Ayim-Akonor (2020)(Ayim-Akonor et al., 2020)	**		*	*	**	***		**	*		12
Ben Hamed (2021)(Ben Hamed et al., 2021)	*		**		*	***		**	*	*	11
Bonney (2012)(Bonney et al., 2012)	**		*		***	*		**	*		10
Byarugaba (2016)(Byarugaba et al., 2016)	**		**		***	***		**	*		13
Dia (2013)(Dia et al., 2013b)	**		**		***	**		**	*	*	13
El Moussi (2013a) (El Moussi et al., 2013a)	**		**	*	***	*		**	**	*	14
El Moussi (2013b) (El Moussi et al., 2013b)	**		**	*	***	*		**	**	*	14
El Moussi (2013c)(El Moussi et al., 2013c)	**		**	*	***	**		**	**		14
El Rhaffouli (2013) (El Rhaffouli et al., 2013)	**		**	*	***	*		**	*	*	13

Gachara (2011)(Gachara et al., 2011)	**		**	*	*	*		*	*	*	10
Gachara (2014)(Gachara, 2014)	**	**	**	*	***	***	*	*	*	*	17
Gachara (2016)(Gachara et al., 2016)	**	**	**	*	***	***	*	*	*	*	17
Meseko (2015)(Meseko et al., 2015)	**		**	**	***	**		*	*		14
Meseko (2019)(Meseko et al., 2019)	**		**		***	***		*	*	*	13
Monamele (2019)(Monamele et al., 2019)	**		**	*	***	**		*	*	*	13
Nakoune (2013)(Nakouné et al., 2013)	**		*		**			***	*	*	10
Nelson (2014)(Nelson et al., 2014)	**		**	**	***	**		***	**	*	17
Opanda (2020)(Opanda et al., 2020)	**		**	*	***	*		*	*	*	12
Orelle (2012)(Orelle et al., 2012)	**		**	*	**	**		**	*	*	13
Pascalis (2012)(Pascalis et al., 2012)	**		**	*	*	***		**	**	*	14
Quiliano (2013)(Quiliano et al., 2013)	**		**	**	***	*		***	**	*	16
Valley-Omar (2015)(Valley-Omar et al., 2015)	**		*	*	*	*	*	***	**	*	13
Venter (2012)(Venter et al., 2012)	**		**	*	***	**		**	**	*	15
**H3N2 viruses**											
Aboualy (2018)(Aboualy et al., 2018)	**		**		*	**		*	**	*	11
Besselaar (1996)(Besselaar et al., 1996)	**		**		***	*		*	*	*	11
Besselaar (1999)(Besselaar et al., 1999)	**		**		***	*		**	**		12
Besselaar (2004)(Besselaar et al., 2004)	**		**	*	*	*		*	*		9
Bulimo (2008)(Bulimo et al., 2008)	**		**		**	*		**	*	*	11
Bulimo (2012)(Bulimo et al., 2012b)	**		**	*	*	**		***	*	*	13
Byarugaba(2011)(Byarugaba et al., 2011)	**		**	**	***	***		***	*		16
El Moussi (2014)(El Moussi et al., 2014)			**		*	*		**	**	*	9
Kaira (2011)(Kaira, 2011)	**		**	**	*	***		***	*	*	15

Kleynhans (2019)(Kleynhans et al., 2019)	*		*	*	*	*		**	**		9
Lemey (2014)(Lemey et al., 2014)	**		***	**	***	*	*	***	**		17
McAnerney (2015)(McAnerney et al., 2015)	**		**	*	*	*		**	**	*	12
Monamele (2017)(Monamele et al., 2017)	**		**	*	***	**		*	*	*	13
Njifon (2019)(Njifon et al., 2019)	**		**	*	***	**		*	*	*	13
Nyang'au (2020)(Nyang'au et al., 2020)	**		***	**	***	*		*	*	*	14
Owuor (2020)(Owuor et al., 2020)	**		**	*	***	*		**	*		12
Westgeest (2014)(Westgeest et al., 2014)			***	**	***	***		***	*	*	16
WHO (2003)(WHO, 2003)	**		**		*	**		*	*	*	10
**H1N1 and H3N2 viruses**											
Barakat (2011)(Barakat et al., 2011)	**		**	*	***	*		**	*	*	13
Barr (2010) (Barr et al., 2010)	**		**		**	**		*	*		10
Besselaar (2001)(Besselaar et al., 2001)	**		**	*	***	*		*	*	*	12
Chan (2010)(Chan et al., 2010)	**		***	**	***	**		***	*	*	17
Deyde (2007)(Deyde et al., 2007)	**		**	*	***	**		*	*		12
Heraud (2012)(Heraud et al., 2012)	**		**	*	***	**		*	*	*	15
Niang (2012)(Niang et al., 2012)	**		**	*	***	**		**	*	*	14
**H1N1pdm09 and H3N2 viruses**											
Ait-Aissa et al 2018(Ait-Aissa et al., 2018)	**		**		***	*		*	*	*	11
Al Khatib (2019)(Al Khatib et al., 2019)	**		*	**	***	**		*	*	*	12
Barr (2014)(Barr et al., 2014)	**		**	*	**	***		*	*		12
Bulimo (2012)(Bulimo et al., 2012c)	**		**	**	**	*		***	*	*	14
Kavunga-Membo (2018)(Kavunga-Membo et al., 2018)	**		**		**	*		**	*		10

Klimov (2012)(Klimov et al., 2012)	**		***	**	***	***		*	*		15
Mackenzie (2019)(Mackenzie et al., 2019)	**		*		**	*		**	*	*	10
Nkwembe (2016)(Nkwembe et al., 2016)			**	*	*	*		*	*		7
Owuor (2021)(Owuor, 2021)	**		**	*	***	***		**	*	*	15
Sanou (2018)(Sanou et al., 2018)	**		*	*	***	*		**	*	*	12
Soli (2019)(Soli et al., 2019)			**	**	***	**		***	*	*	14
Soliman (2020)(Soliman et al., 2020)	**		*	*	***	**		*	*	*	12
Tivane (2018)(Tivane et al., 2018)	**		*	*	**	**		**	*	*	12
Valley-Omar (2018)(Valley-Omar et al., 2018)	**		**	*	**	*		***	**	*	14
**H1N1, H1N1pdm09 and H3N2 viruses**											
Treunicht (2019)(Treurnicht et al., 2019)	**		**	*	***	*		*	*		11
Wadegu (2016)(Wadegu et al., 2016)	**		**	*	***	**		*	*	*	13

Table S7: Detailed study sampling bias assessment

Study (Reference)	Can the study be generalized to the study population i.e., all-round surveillance programme?	How many eligible viruses were sequenced and had epidemiological data?
	**H1N1 viruses**	
Besselaar (2008)(Besselaar et al., 2008)	Study nested in the National Institute of Communicable Diseases (NICD) active sentinel surveillance program collected 45 consecutive samples during a 3-month period between May and July 2008 in all 9 provinces across South Africa; the 23 viruses isolated in May-June were analysed phenotypically while all 45 viruses were tested for presence of the H274Y oseltamivir resistance marker using both rapid PCR and sequencing	Sequenced all 45 A(H1N1) viruses isolated in the May-July 2008 period. All samples had data on location and date of sampling
Bulimo (2012)(Bulimo et al., 2012a)	Study nested in sentinel-site based surveillance system for influenza collected a total of 5898 nasopharyngeal swab specimens during the 23-month period between January 2007- November 2008 from eight (8) hospitals across Kenya; 1 provincial hospital in Kisumu and 7 district hospitals in the cities of Nairobi, Mombasa, Malindi, Isiolo, Kericho, Kisii, and Alupe; randomly selected 72 isolated stratified by month and location of sampling; selected 42 H1N1 positives out of 72 viruses for genotypic analysis	Sequenced HA1 genes for all the 42 H1N1 positives; All samples had data on location and date of sampling
Hurt (2009) (Hurt et al., 2009)	Study nested in the WHO global influenza Surveillance network did both phenotypic and genotypic analysis of 264 viral isolated from 10 countries from Oceania, South East Asia and South Africa; sampled only Australia (111), Macau (34), Malaysia (34), New Caledonia (7), New Zealand (4), Philippines (11), Singapore (10), South Africa (26), Taiwan (5) and Thailand (22)	Screened all 264 samples for H274Y using pyrosequencing. Sequenced only a subset of the 264 positive viruses which showed reduced susceptibility to Oseltamivir and Peramivir in the phenotypic analysis. All samples had demographic data on location and date of sampling.
Njouom (2010)(Njouom et al., 2010)	Study nested in the national influenza Surveillance network did both phenotypic and genotypic analysis of 26 influenza positive viruses [10 H3N2, 10 H1N1, 6 B] A(H1N1) from 7 sentinel sites across Younde, Cameroon	Sequenced all the 10 A(H1N1) positive viruses. All viruses had demographic data on location and date of sampling.
Dia (2013)(Dia et al., 2013a)	Study nested in an influenza surveillance network did both phenotypic and genotypic analysis of 86 and 10 H1N1 viruses, respectively isolated between July and September 2008 from suburbs in Dakar, Senegal; included 27 viruses earlier isolated in 2007 from Dakar	Only a subset 10 (M2, NA) and 5 (HA1) of the 86 H1N1 viruses were sequenced. All samples had data on location and date of sampling
	**H1N1pdm09 viruses**	
Adeola (2019)(Adeola et al., 2019)	Independent survey study did syndromic sampling of 132 asymptomatic swine (for 14 months, Jan 2014- March 2015) and 87 swine handlers with ILI (for 4 months, Dec 2015-March 2016) in pig farms and Abattoirs in Ibadan (Nigeria, n=68) and Kumasi (Ghana, n=19). Included unknown number of Africa and global human (2009-2014) and swine (2011-2018) viral MP sequences from GenBank	Sequenced all (3) human samples positive for H1N1pdm09 [Nigeria (1) and Ghana (2)]. Detected H3N2 viruses in both humans and swine but did not report on them. Sequenced viral samples had epidemiological data
Aspinall (2013)(Aspinall et al., 2013)	Independent trial study that collected consecutive samples from 54 patients during 1-year period from 2 sites (not described) in South Africa. Treated all patients with oseltamivir at day 1 and day 5 and followed up at 10 after treatment.	Sequenced viruses from all 44 of the 52 patients who tested positive for H1N1pdm09 virus. All viral samples had demographic and clinical data.
Ayim-Akonor (2020)(Ayim-Akonor et al., 2020)	Independent study collected consecutive viruses from 12,000 health swine and 99 asymptomatic swine farmers from 87 farms for 2 seasons [dry (April-July 2016) and rainy (Dec 2016-Feb 2017)] in Ashanti region of Ghana. Included 16	Sequenced only a subset (8) of 17 swine H1N1pdm09 viruses with PCR <25. Reported phylogenetic clustering results for HA and NA alone even though they had WGs

	Ghanaian human and both human and viral sequences (available on GISAID) sampled from the rest of Africa and globe in the same season (2016-2017).	for both swine (sequenced) and human viruses (GISAID). Samples had all the epidemiological data.
Ben Hamed (2021)(Ben Hamed et al., 2021)	Independent study collected consecutive viruses from 31 H1N1pdm09 positive patients aged (3 months-80 years old) with SARI for a period of 12 months (2017- 2018) in Monastir region, Tunisia, localized in the coastal region of Mediterranean Sea in North Africa with Sebkha islands, creating a major site for migratory birds. Included global N1 sequences sampled in 2010-2011.	Sequenced 7 of the 31 H1N1pdm09 positive samples. The 7 viruses were from patients with fatal and severe cases with ≤5 days of infection and the best PCR NA bands sampled in Nov 2017-Feb 2018. Samples had all the epidemiological and clinical data
Bonney (2012)(Bonney et al., 2012)	Study nested in the national influenza surveillance system compromising of 22 health facilities across Ghana consecutively collected samples from children < 11 years old with ILI for a 3-year period (Jan 2008- Dec 2010)	Sequenced a subset (13) of all the 342 H1N1pdm09 positive samples. Sequences had demographical and clinical data
Byarugaba (2016)(Byarugaba et al., 2016)	Study nested in a hospital-based sentinel surveillance system collected consecutive samples from ILI patients of all ages for a 23-month period (July 2009 – May 2011). The surveillance consisted 4 hospital sites: Mulago National Referral Hospital (central), Jinja Regional Referral Hospital (eastern), Bugiri District Hospital (eastern), and Gulu Regional Referral Hospital (northern)	Sequenced viral whole genomes from a subset (19) of 73 H1N1pdm09 positive samples collected in the 2009-2011 seasons. All sequences had demographic data, location, and year of sampling
Dia (2013)(Dia et al., 2013b)	Study attached to the national sentinel surveillance that collects consecutive isolates during 17-month period from 7 Senegalese sites (N=7). Other sequenced samples from Cape Verde (1), Guinea (0), and Mauritania (6) were collected in context of the pandemic from only urban clinics, 13 of the sequenced strains were collected in 2010; such low numbers limited characterization of viruses circulating in well-defined seasons and geographical region	Sequenced a subset (14) of the 503 H1N1pdm09 positive samples. All viral samples had demographic but not clinical data (date, location, gender, and age).
El Moussi (2013a)(El Moussi et al., 2013a)	Study nested in a viroloigcal surveillance network sampling community, hospitalised, and fatal cases collected consecutive samples (3982) for 2 years (2009-2011) from the ILI and SARI patients of all ages in Tunisia.	Sequenced HA genes from a subset (50) out of the 3982 H1N1pdm09 positive samples; 42 from severe and fatal cases and 8 from mild clinical cases. All sequenced samples had epidemiological or demographic and clinical data
El Moussi (2013b)(El Moussi et al., 2013b))	Study nested in a sentinel centre and a hospital collected consecutive samples for 21-month period between May 2009 and December 2011 from ILI patients of all ages in Tunisia	Sequenced partial HA genes from a subset (50) of all collected H1N1pdm09 positives; sequenced 23 viruses from 2009-2010 and 27 viruses from 2010-2011 seasons; All sequenced samples had demographic and clinical data
El Moussi (2013c)(El Moussi et al., 2013c)	Study nested in an influenza surveillance programme comprising 268 primary care centres in 24 governorates covering 2.7% of general Tunisian population. Programme collected consecutive samples for 4-year period [2008 - 2011] from ILI and SARI patients of all ages.	Sequenced HA and NA genes from a subset (50) of 3982 H1N1pdm09 positive viruses collected in both the 2009-2010 and 2010-2011 flu seasons. All sequenced samples had epidemiological and clinical data.
El Rhaffouli (2013)(El Rhaffouli et al., 2013)	Independent surveillance initiated at a hospital in Rabat collected consecutive samples from 1183 ILI patients during 2-years period from Rabat. Sequenced 11 strains collected in 2009, 5 (2010) and 2 (2011). Included 14 online (GenBank) sequences collected from another Moroccan city, Casablanca	Sequenced a subset (22) of the 368 H1N1pdm09 cases sampled. All sequenced samples had epidemiological or demographic and clinical data
Gachara (2011)(Gachara et al., 2011)	Independent study sampled consecutive viruses during 6-month period from all provinces across Kenya. Sequenced 31 viruses collected in 2009 from Kenya; included viruses from Nigeria (3), Senegal (4), Ethiopia (2), and Mali (1)	Sequenced a subset (31) of all H1N1pdm09 positive viruses collected across Kenya. All viral samples had data on date and location of sampling.
Gachara (2014)(Gachara, 2014)	PhD Study nested in the pandemic outbreak surveillance system at the Kenyan NIC collected consecutive samples for a 13-month period [July 2009 (early phase), August- Oct 2009 (peak), and Nov 2009- Aug 2010 (late phase)] from	Sequenced whole genomes from as subset (40) of the collected 369 viruses that tested positive for H1N1pdm09

	15 sentinel sites around Kenya; included 320 viruses from the NCBI H1N1 Flu Influenza Resource Database sampled around the globe (Africa, UK, USA and China) during the same year.	infection. All sequences had information on location and year of sampling.
Gachara (2016)(Gachara et al., 2016)	Study nested in the global influenza pandemic response system collected consecutive samples from ILI patients of all ages for a 13-month period [July 2009 (early phase), August- Oct 2009 (peak), and Nov 2009- Aug 2010 (late phase)] from hospitals doing routine diagnostic treatment, sentinel hospitals, and medical facilities across Kenya	Sequenced a subset (40) of the 369 H1N1pdm09 positive samples collected. Sequenced samples had data on year and location of sampling
Meseko (2015)(Meseko et al., 2015)	Independent study downloaded 115 HA and 75 NA genes of African H1N1pdm09 viruses from Genbank and GISAID based on the gene segment length, country of origin, and host species (human and swine); duplicated sequences and short segments were excluded. Analysed only 12 and 46 human viruses for mutations and phylogenetic analysis, respectively, out of all downloaded. Sequences analysed were isolated from various African countries including: Tunisia Nigeria, south Africa, Cameroon, Angola, Uganda, Ghana, Algeria, Djibouti, Egypt, Ethiopia, Ivory Coast, Zambia, Mali, Togo. Each country had 1 representative sequence analysed. Included 4 swine viruses sampled in Cameroon (2010), Nigeria (2011), Kenya (2011), and Togo (2013).	Analysed a subset (46) of the retrieved sequences. Analysed sequences had data on location and year of sampling
Meseko (2019)(Meseko et al., 2019)	Independent study collected consecutive viruses from 227 swine with influenza-like signs during a 24-month period (July 2010-June 2012) from a peri-urban zone in Lagos, southwestern Nigeria. Included unknown number of African human (Nigeria, Ghana, Cameroon, Kenya, Mali, Ivory Coast, and Seychelles) and global human, avian and swine viral whole genomes from GenBank and GISAID	Sequenced a subset (12) of 18 swine H1N1pdm09 viruses that were successfully isolated or cultured. However, 31 swine tested H1N1pdm09 positive by RT-PCR. Did not mention the number of African or global human H1N1pdm09 viruses included in the study. Samples had data on the year and location of sampling
Monamele (2019)(Monamele et al., 2019)	Study nested in an influenza surveillance system collected consecutive samples for 2·5 years from 2 sentinel sites in Cameroon; included 22 Cameroon viruses from GISAID collected between the same period; analysed 39 viruses in total; collected in 2014 (2), 2015 (30), and 2016 (7)	Sequenced a subset (23) from all the 122 influenza positives samples observed in the surveillance. Analysed sequences had data on location and year of sampling
Nakoune (2013)(Nakouné et al., 2013)	Independent surveillance network initiated in Bangui collected consecutive samples (329) during a 1-year period from Bangui and 3 rural areas in Central African Republic (CAR); only 5 samples were positive for H1N1pdm09 collected and these were collected between 26 July- 22 October 2010	Sequenced partial HA genes (253bps) from all the 5 H1N1pdm09 positive samples collected. All sequenced samples had epidemiological or demographic and clinical data
Nelson (2014)(Nelson et al., 2014)	Independent study downloaded and analysed 8712 and 7644 full-length HA (H1) and NA (N1) sequences of H1N1pdm09 viruses collected globally from GISAID database; of these 299 were from 18 African countries; 3 nations in North Africa (Algeria, Morocco, and Egypt), 7 West Africa (Burkina Faso, Cameroon, Côte d'Ivoire, Ghana, Niger, Nigeria, and Senegal), 7 East Africa (Djibouti, Ethiopia, Kenya, Madagascar, Tanzania, Uganda, and Zambia), and South Africa	Analysed all downloaded full-length HA and NA sequences. All sequences had data on location and year of sampling
Opanda (2020)(Opanda et al., 2020)	Study nested in a country-wide human respiratory viruses sentinel surveillance network that sampled respiratory swabs from both ILI and SARI patients of all ages in of 7 district hospitals [Mbagathi, New Nyanza, Malindi, Isiolo, Mombasa, Port Reitz, and Kericho] around Kenya for 4 years 2015 (2), 2016(2), 2017 (2), and 2018 (29).	Sequenced all (38) H1N1pd09 positive viral viruses. Viruses had data on country and date of sampling but did not specify city or site of sampling.
Orelle (2012)(Orelle et al., 2012)	Study nested in the national sentinel surveillance network that collected consecutive viruses during 7-month period from 24 sites (hospitals and clinics) from 17 of the total 22 health regions across Madagascar; H1N1pdm09 positive cases confirmed from only 16 sites; sufficient national geographical coverage	Sequenced a subset (26) of the 1016 H1N1pdm09 positive viruses. All sequenced samples had epidemiological or demographic and clinical data
Pascalis (2012)(Pascalis et al., 2012)	Independent cohort study sampled 1,196 swabs from 443 individuals in 125 households following ILI symptoms alerts; these were collected in a 4-month period during the 2009 pandemic; households selected to represent a wide range of geographical locations in Reunion Island. Selected samples for sequencing to reflect epidemiological and temporal dynamics of the epidemic in the cohort.	Sequenced viruses from a subset (28) of the 101 swabs positive for H1N1pdm09 viruses. All sequenced samples had demographic and clinical data

Quiliano (2013))(Quiliano et al., 2013) Quiliano (2013) (Quiliano et al., 2013)	Independent global study retrieved 3740 NA protein sequences from the Influenza virus sequence database, these were from H1N1pdm09 strains isolated globally for a 2-year period. Number of sequences per continent included Africa (59), America (2298), Asia (521), Oceania (89), and Europe (772); specific African regions from which strains were sampled not mentioned; sample number justified since all available full-length protein sequences were analysed; the study did not compute frequency of each type of AAS based the number of NA sequences readily available in the database at that year not taking into account differences at a continental level, which could have created a reporting bias	Analysed only full-length protein sequences. Analysed sequences had data on year and location of sampling
Valley-Omar (2015)(Valley-Omar et al., 2015)	Independent hospital-based study sequenced 18 H1N1pdm09 viruses from frozen samples collected from children by the National Health Laboratory in Cape Town, South Africa. Samples were collected for 4 months (April-July 2011) from 4 hospitals; RXH (14), Groot Schuur Hospital (1), 2-military hospital (1) and Somerset hospital (2).	Sequenced partial HA genes from all the 18 H1N1pdm09 positive samples collected during the 4-months period; All patient samples had data on year of sampling and clinical presentation but only 9 patients had all the epidemiological data
Venter (2012)(Venter et al., 2012)	Study nested in multiple (3) influenza surveillance systems that collected consecutive viruses from 16707 ILI and SARI patients of all ages during an 18-month period [July 2009-Dec 2010] from 246 sentinel health centres and 15 hospitals in approximately 9 provinces across South Africa.	Sequenced a subset 72 HA and 118 NA out of the 1480 H1N1pdm09 positives sampled. Samples data on year and date of sampling and clinical data
**H3N2 viruses**		
Aboualy (2018)(Aboualy et al., 2018)	Study nested in a national influenza surveillance programme collected 480 consecutive samples from ILI outpatients of all ages during a 3-month period (Oct- Dec 2014) from 8 hospitals in Egypt	Selected and successfully sequenced a subset (4) of all the influenza 84 H3N2 positive samples collected during the sampling period. Sequences had data on location and year of sampling
Besselaar (1996)(Besselaar et al., 1996)	Study nested in a viral watch programme obtained 74 (42 H3N2 and 32 B) viruses collected during a 15-month period (June 1993 – September 1994) from South Africa. These were from ARI patients from various clinics and hospitals in SA.	Sequenced a subset (9) of the 42 H3N2 influenza positives. Sequenced samples had data on location and year of sampling
Besselaar (1999) (Besselaar et al., 1999)	Study sampled viruses from 404 patients of all ages (ARI patients of all ages and infants with SARI) for a 2-year period (1997-1998) through both an active surveillance programme and routine diagnosis system at various sites in South Africa	Number of sequenced viral samples not not described but reported genomic results for a subset (15) of the 139 H3N2 viruses that were successfully isolated. Sequences had demographical and clinical data
Besselaar (2004)(Besselaar et al., 2004)	Study collected 684 consecutive samples from a police residential college in Pretoria (closed community, 25th May-7th June 2003) and 34 sporadic cases (13 May – 30 June 2003) from a surveillance programme consisting of 14 centres (general medical practitioners, paediatric outpatient departments at hospitals, a university clinic, two school clinics, two retirement homes, and the staff clinic at NICD) in surrounding areas (Johannesburg, Middleburg, and Vanderbijlpark).	Sequenced all 50 H3N2 positive samples collected from all areas during the sampling period. Sequences had data on location and year of sampling.
Bulimo (2008) (Bulimo et al., 2008)	Study nested in an active multiple-institute influenza surveillance system collected 1014 consecutive samples from ILI Outpatients with ILI with age of 2 months and above during a 9-month period (July 2006- April 2007) at active hospital-based sentinel sites: Mbagathi, New Nyanza, Malindi, Isiolo, Mombasa, Port Reitz, and Kericho in Kenya	Sequenced viruses from all (9) samples that tested positive for H3N2 during the 9 months of sampling. Sequences had clinical, demographic and influenza vaccination data.
Bulimo (2012)(Bulimo et al., 2012b)	Study nested in a surveillance system collected 708 consecutive samples from ILI patients of > 2 months of ages during 3-month period (October-December 2010) at 8 sentinel sites (hospitals); Cities: Kisumu, Nairobi, and Mombasa and towns: Malindi, Isiolo, Kericho, Kisii, and Alupe	Sequenced all (32) H3N2 positives of the total 708 samples collected through the surveillance. Sequences had data on location and year of sampling

Byarugaba(2011)(Byarugaba et al., 2011)	Study nested in a routine influenza surveillance system collected 932 consecutive samples from ILI out patients aged 6 months and above for a 2-year period (1st Oct 2008-30th sept 2009) from two sites, Mulago (urban) and Kayunga (rural) hospitals in Uganda.	Sequenced all the 59 samples that tested positive for influenza H3N2 out of the 932 total samples collected during the 2-year period. Sequences had data on location and year of sampling
El Moussi (2014)(El Moussi et al., 2014)	Study collected clinical samples from patients of all ages with mild, severe and fatal cases of confirmed H3N2 infection between 29th January and February 2013. Total number of samples collected not mentioned.	Sequenced HA genes from 5 H3N2 influenza positive samples. Not clear whether the 5 was a subset or all the positive samples collected. Sequences had demographical and clinical data
Kaira (2011)(Kaira, 2011)	Study nested in a routine influenza surveillance system collected 450 consecutive samples from ILI out patients aged 6 months and above for a 3-month period (Oct – Dec 2008) from two sites, Mulago (urban) and Kayunga (rural) hospitals in Uganda.	Sequenced all the 50 H3N2 influenza positives out of the 450 samples collected during the 3-month period. Sequences had demographic data, data on location and year of sampling
Kleynhans (2019) (Kleynhans et al., 2019)	Independent cohort study collected 38 respiratory samples from students with ILI in grade 8-12 and bridge year in July 2016 before and during an outbreak in a boarding school (closed population) in the East Cape province in South Africa. 4 samples were collected before the outbreak; Included other 42 South Africa H3N2 virus sequences from GISAID collected between 19th May and 8th August 2016 (collected from other provinces WCP, GP, KNP, FSP)	Sequenced a subset (19) of the 38 H3N2 positive samples collected during the sampling period but analysed only 14 sequences from the ECP outbreak. Sequences had demographical and clinical data
Lemey (2014) (Lemey et al., 2014)	Independent global study analysed 1529 sequences of global H3N2 viruses collected for 6-year period (2002- 2007); These included 31 from Africa (Algeria, Egypt, Madagascar, South Africa and Saudi Arabia) and 1,498 from the rest of the world (Europe, Asia, Oceania USA)	Global study analysed 1529 [Africa (31) and rest of the world (1,498)] H3N2 virus sequences. Sequences had data on location and year of sampling
McAnerney (2015)(McAnerney et al., 2015)	Study nested in a sentinel surveillance programme collected 834 consecutive samples from ILI patients of all ages during a 5-month period (May-Sept 2014) at 65 practices in 8 of the 9 provinces in South Africa.	Sequenced a subset 34 [vaccinated (10) and unvaccinated (24)] of the 472 influenza positives (or 336 H3N2 positives) collected. Sequences had clinical, demographic, and influenza vaccination data collected.
Monamele (2017)(Monamele et al., 2017)	Study nested in an influenza surveillance system sampled H3N2 viruses during 2.5-year period (Jan 2014 -June 2016) from 12 sentinel sites in Cameroon. Samples per year 2014 (n=6), 2015 (n=17), and 2016 (n=12)	Selected and sequenced a subset (35) of the sampled viruses. Sequenced samples had data on location and year of sampling
Njifon (2019)(Njifon et al., 2019)	Study nested in an influenza surveillance system sampled H3N2 viruses during 2.5-year period (Jan 2014 -June 2016) from 6 sentinel sites in Northern Cameroon; samples per year 2014, (1) 2015 (5) and 2016 (10); included 35 sequences from Southern Cameroon sampled in 2014-2016	Selected and sequenced a subset (16) of the sampled viruses. Sequences had data on location and year of sampling
Nyang'au (2020)(Nyang'au et al., 2020)	Independent study analysed 115 H3N2 virus HA1 gene sequences collected during a 7-year period (2007-2013) from Kenyan hospitals through the human respiratory virus surveillance program, within the Kenya Medical Research Institute (KEMRI)	Analysed all 115 available sequences. All sequences had data on the location and year of sampling
Owuor (2020)(Owuor et al., 2020)	Study nested in two hospital-based surveillance systems (KCH for pneumonia and KHDSS for influenza) collected 11558 from both in and outpatients of all ages for 9-year period (Jan 2009 – March 2017 (9 years)); the health centres (sentinel sites) were all located in Kilifi a coastal town in Kenya.	Sequenced a subset (152) of the 292 samples that were influenza A positive; of these 101 were H3N2 positives and 41 H1N1pdm09 positives. Analysed only H3N2. Sequences had demographical and clinical data
Westgeest (2014)(Westgeest et al., 2014)	Study nested to the global WHO influenza surveillance network sequenced H3N2 viruses from previously collected clinical samples and viral isolates for a period of 40 years (1968-2011). Sequenced viral samples spanned Asia, America, Europe, and South Africa (n=3). Included online WGs and PGs inclusive of 3 viruses from South Africa.	Sequenced all 284 viruses that were successfully cultured by a pervious study. Sequenced samples had data on the location and year of sampling

WHO (2003)(WHO, 2003)	Study nested in a surveillance system undertook an independent retrospective morbidity survey and collected data from patients of all ages presenting at 10 health posts. it identified 792 ARI patients from whom they chose 6 samples for serological and genomic analysis as described by (Schoub et., al 1986)	Sequenced a subset (4) of the 792 ARI patient samples collected between week 49-50. Sequences had data on location and year of sampling and clinical symptoms
**H1N1 and H3N2 viruses**		
Barakat (2011)(Barakat et al., 2011)	Study nested in the national sentinel influenza surveillance system collected 673 consecutive samples during a 3-year period (1996-1998) from ILI patients of all ages at 8 sites in Morocco (Rabat, Kenitra, Casablanca, Marrakech, Agadir, Fes, Oujda, and Tangier); number of samples collected per year: 1996 (110), 1997 (228) and 1998 (335)	Sequenced a subset 29 (21 H3N2, 5 H1N1, and 3 B) of the total 107 viruses that were successfully isolated by culturing. Sequences had demographic and clinical data.
Barr (2010) (Barr et al., 2010)	Study nested to the WHO global influenza surveillance network analysed seasonal H1N1 and H3N2 viral isolates consecutively sampled between Sept 2008 and Feb 2009 from various laboratories worldwide and submitted to the World Health Organization Collaborating Centers.	Included only 1 H1N1 and 2 H3N2 viral strains from Africa in the phylogenetic analysis. Sequences had demographic and clinical data.
Besselaar (2001)(Besselaar et al., 2001)	Study nested in an active surveillance program and routine diagnosis systems sampled viruses from ARI and SARI patients of all ages for a 3-year period (1997-1999) in various sites in South Africa	Number of viral samples sequenced undescribed but reported genomic results for 7 H1N1 and 19 H3N2 viruses. Sequences had data on location and year of sampling
Chan (2010)(Chan et al., 2010)	Independent study applied a probabilistic model on >6000 HA, NA and HA1 online sequences sampled for a period of 6 years (2004-2009) form Asia, Europe, America, Oceania and Africa (Kenya) to characterize their migration between regions (source-sink)	Analysed all available complete HA and NA and HA1 sequences available through NCBI at the time. These included ~100 HA1 sequences sampled in Kenya. All samples had data on location and date of collection
Deyde (2007)(Deyde et al., 2007)	Study nested to the WHO global influenza surveillance network analysed 3146 H3N2 and 619 seasonal H1N1 viruses consecutively sampled between oct 2004 and September 2006 from various laboratories worldwide and submitted to the World Health Organization Collaborating Center for Surveillance, Epidemiology and Control of Influenza at the CDC (Atlanta). 13 of these viruses were sampled in Africa (6 H3N2 viruses from both Egypt and South Africa, and 7 H1N1 viruses from Egypt alone)	Screened all 3,765 virus viruses for adamantane resistance using pyrosequencing. Sequenced M2 genes from 20 viruses to confirm results from pyrosequencing. Sequenced complete MP genes for 57 [seasonal H1N1 (10), H3N2 (47) viruses for phylogenetic analysis; Sequenced HA1 genes from unknown number of H3N2 and 72 H1N1 viruses, respectively. Phenotypic analysis done for only 11 H1N1 viruses. All samples had data on location and date of sampling
Heraud (2012)(Heraud et al., 2012)	Study nested in national public health sentinel surveillance systems collected a total of total of 8312 samples from 5 countries—Cameroon, Côte d'Ivoire, Madagascar, Niger, and Senegal. Sentinel sites consisted both public and private clinics urban or semi-urban areas. Selected 113 AH1N1 and AH3N2 viruses for phenotypically (antigenic) analysis. Analysed sequences for 113 AH1N1 viruses (2008-2009) [Cameroon (n=28), Ivory Coast (n=24), Madagascar (n=21), Senegal (n=33), and Seychelles (n=7)] and 151 AH3N2 viruses (2008-2010) [Cameroon (n=36), Ivory Coast (n=25), Madagascar (n=42), Niger (n=12), and Senegal (n=36)].	Sequenced HA1 and NA genes from 113 H1N1 and 115 H3N2 viruses. All samples had data on location and date of sampling
Niang (2012)(Niang et al., 2012)	Study nested in the national influenza surveillance centre (NIC) network of Senegal collected a total of 9177 samples during a 14-year period (January 1996 – December 2009) from ILI outpatients of all ages at 13 sentinel health centres located in Dakar city and 2 rural sites.	Sequenced a subset 36 (24 H3N2, 9 H1N1, 3 H1N2) of the total 958 influenza A positives. All samples sequenced had demographic and clinical data
H1N1pdm09 and H3N2 viruses		

Ait-Aissa (2018)(Ait-Aissa et al., 2018)	Study nested in the national sentinel surveillance network that collected consecutive samples during 6-years period from 9 provinces (in the North centre and North East regions of Algeria; selected 378 samples out of 1460 positives for viral isolation. Sequenced 11 viruses (3 H1N1pdm09, 6 H3N2 and 2 B viruses) with high IC_{50} values; sufficient national geographical coverage but should have sequenced all 378 to capture other important mutations contributing to variation in NA inhibition	Only a subset (11) of the selected 378 positive cases were sequenced and analysed genotypically respectively. All viral samples had demographic and clinical data
Al Khatib (2019)(Al Khatib et al., 2019)	Independent study retrieved 1226 online sequences retrieved from the Influenza Research Database (IRD) collected during a period of 9-years (January 2009 and December 2017) from Middle East and North Africa (MENA) regions; Middle East (Saudi Arabia, Iran, Oman, Kuwait, Bahrain, Turkey, Jordan) and North Africa (Tunisia, Egypt). A total of 512 H1, 239 H3, 343 N1, and 132 N2 gene sequences were analysed	Retrieved and analysed a non-specified number of sequences from North Africa. All results except the phylogenies were not reported distinguishably between Middle East and North Africa. Analysed sequences had data on location and year of sampling
Barr (2014)(Barr et al., 2014)	Study nested to the WHO global influenza surveillance network analysed H1N1pdm09 and H3N2 viral isolates consecutively sampled between Sept 2012 and Feb 2013 from various laboratories worldwide and submitted to the World Health Organization Collaborating Centers.	Study had 379 H1N1pdm09 and 872 H3N2 including 226 Africa strains sequences (35 H1N1pdm09, 115 H3N2, and 76 B) but reported results for just a subset (4 H1N1pdm09 and 5 H3N2). Analysed sequences had data on location and year of sampling
Bulimo (2012)(Bulimo et al., 2012c)	Study nested in the national influenza surveillance network collected consecutive samples from patients of over 2 months with acute respiratory illnesses (ARI) during the 2010-2011 influenza season from 7 district hospitals (Mbagathi, New Nyanza, Malindi, Isiolo, Mombasa, Port Reitz, and Kericho) across Kenya. The hospital sites were chosen based on their geographical regions and population demographics.	Sequenced HA1 genes from all influenza positive samples collected during the sampling period including 27 H1N1pdm09, 19 H3N2 and 16 B viruses. Sequences had data on location and year of sampling
Kavunga-Membo (2018)(Kavunga-Membo et al., 2018)	Study nested in an influenza sentinel surveillance system collected 2376 consecutive samples from ILI and SARI patients of all ages during a 1-year period (2015) at 11 urban and rural sentinel sites in five (Kantaga, Maniema, Kasai-oriental, Bas-congo and Kinshasa) of the 11 provinces in Democratic Republic of Congo. Sites selected based on easy accessibility to patients, high qualification of staff and adequate laboratory facilities.	Sequenced a subset (undescribed) of the 218 influenza A positive samples collected. Sequences had data on age, location, and year (year) of sampling and clinical symptoms.
Klimov (2012)(Klimov et al., 2012)	Study nested to WHO global influenza surveillance network analysed online (GISAID) 4400 viruses from clinical samples and viral isolates (from patients of all ages) sampled in Africa, Asia, America, Europe, Oceania through the WHO-National influenza centres and laboratories from 2009-2011. The Africa viruses included 114 H1N1pdm09 [Algeria (1), Cameroon (2), Ivory Coast (2), Egypt (4), Ethiopia (4), Ghana (30), Kenya (19), Madagascar (7), Niger (4), Nigeria (6), Senegal (14), South Africa (6), Tanzania (4), and Uganda (1)] collected from 1st April 2010-23rd May 2011, and 112 Africa H3N2 [Algeria (n=2), Cameroon (n=12), Ivory Coast (n=11), Ghana (n=19), Kenya (n=11), Madagascar (n=24), Morocco (n=1), Nigeria (n=2), South Africa (n=21), Tanzania (n=8) and Tunisia (n=1)] collected from 5 Dec 2010 -26 May 2011.	Did genomic analysis on a subset (~1600 H1N1pdm09 and ~1000 H3N2) of viral whole- and partial genomes out of the 4400 global viruses sampled in 2009-2011 and available on GISAID at a time. Sequences had data on time and location of sampling. Not all Africa viral sequences were included in the phylogenies. Specifically, out of the 114 Africa viral H1-HA1 sequences only 7 were included in the HA1 phylogeny, and of the 112 H3-HA1 sequences only 12 were included in the HA1 phylogeny
Mackenzie (2019) (Mackenzie et al., 2019)	Study nested in a population-based surveillance study collected consecutive samples during 11-month period from 2-23 months old patients with ALRI and suspected pneumonia; The Basse Health and Demographic Surveillance Systems (BHDSS) consisted of 5 health facilities	Sequenced a subset (16) of the 39 influenza positives (4 H1N1pdm09, 4 H3N2 and 8 B) during the sampling period. All sequenced samples had demographic and clinical data
Nkwembe (2016)(Nkwembe et al., 2016)	Study nested in the National Public health Influenza surveillance system collected 806 consecutive samples from ILI and SARI patients of all ages during a 5-month period (Aug -Dec 2014) at various unnamed sentinel sites in Democratic Republic of Congo (DRC).	Sequenced a subset (20) of the 61 influenza A positive samples. Sequences had data on date and location of sampling

Owuor (2021)(Owuor, 2021)	Academic research study nested to 5 disease surveillance and research programmes (National Influenza Sentinel Surveillance Study (2009-2018), Studying the Pathways of Respiratory virus Disease transmission (SPReD-Kenya) Study (2014-2016), Kilifi County and Referral Hospital (KCH) Study (2009-2018), Kilifi Health and Demographic Surveillance (SPReD-KHDSS) Study (2015-2017), Pneumonia Etiology Research for Child Health (PERCH-Africa) Study (2011-2013)] sampled 798 clinical samples collected from SARI, pneumonia, ARI and lower respiratory tract infection (LRTI) patients of all ages for a period of 10 years (2009-2018) across Kenya. The PERCH-Africa Study sampled viruses from children in The Gambia, Kenya, Mali, South Africa, and Zambia.	Sequenced viruses from all IAV positives [Kilifi (n=66), Kenya (383), and Africa (n=100), consisting of H1N1pdm09 (414) and H3N2 (135) viruses] with sufficient volume but analysed only those with WGs. Included 1, 587 global H1N1pdm09 WGs [Africa (155); Asia (372); Europe (326); North America (356); South America (181); and Oceania (197)] sampled in March 2009-Dec 2018 and 1,571 global H3N2 WGs [Africa (281); Asia (250); Europe (250); North America (250); South America (290); Oceania (250)] sampled in Jan 2014-Dec 2016 downloaded from GISAID. Sequenced sampled had epidemiological and clinical data.
Sanou (2018)(Sanou et al., 2018)	Study nested in the influenza sentinel surveillance system at the national reference laboratory collected 924 consecutive samples from children below 5 years old with ILI and SARI during a 2-year period (January 2014 and Dec 2015) at six health centres in Burkina Faso.	Sequenced a subset 43 (14 H1N1pdm09 and 29 H3N2) of the 124 influenza A positive samples collected. Sequences had demographic and clinical data
Soli (2019)(Soli et al., 2019)	Independent study downloaded available online (NCBI) 102 sequences of viruses (H1N1pdm09, H3N2 and H9N2) collected between 2009 and 2013 from Tunisia: The sequences consisted of HA and NA sequences for all 3 subtypes and PB2, NP and M sequences for o the avian H9N2.	All 102 sequences for PB2, HA, NP, NA and M were analysed. Sequences had data on year and location of sampling
Soliman (2020)(Soliman et al., 2020)	Independent study collected 60 consecutive samples from children ages <5 years old with ILI during a period of 24 months (Jan 2015-Dec 2016) at a paediatric hospital in Aboelreesh, Giza, Egypt. Included 116 H1N1pdm09 (2009-2017) and 82 H3N2 (2006-2017) Egypt viral sequences from GISAID	Selected and sequenced 10 out of the 26 samples positive for IAV. Only 5 (2 H1N1pdm09 and 3 H3N2) of the 10 were successfully sequenced. Sequenced samples had data on location and year of sampling
Tivane (2018)(Tivane et al., 2018)	Study nested in the National Institute of Health (NIH) influenza sentinel surveillance system collected 1140 consecutive samples from children (0-12) years old with SARI during the 2005 influenza season at two hospitals (Mavalene general and Maputo central) located in Maputo City, Mozambique	Sequenced a subset (20) of the 46 influenza A and B positive samples collected. 95% (19) viruses were successfully sequenced and analysed: 12 H3N2, 4 H1N1pdm09, and 3 B. Sequences had clinical and demographical data and year and location of sampling.
Valley-Omar (2018)(Valley-Omar et al., 2018)	Study part of a large Household Transmission Study collected samples from ILI patients including 30 index cases (sampled once at enrolment) and their 107 contact cases (swabbed at enrolment and every after 4 days for 12 days) during a 6-month period (May – October 2013) at 2 peri-urban sites Klerksdorp and Pietermaritzburg in South Africa	Sequenced all (35) samples collected from households with either H1N1pdm09 or H3N2 viral transmissions during the sampling period. Sequences had data on demographic, year and location of sampling and co-infection with HIV.
**H1N1, H1N1pdm09 and H3N2 viruses**		
Treunicht (2019)(Treurnicht et al., 2019)	Study nested in the national influenza surveillance system that collected 15,985 consecutive samples during 7-year period between 2007 and 2013 in nine provinces in south Africa; selected and analysed a portion of influenza A and B positive samples using both phenotypic (antigenic) and genotypic assays	Sequenced only a subset (140) of the 5070 influenza A and B positive samples. Did not sequence any seasonal H1N1 viruses collected between 2007-2008. Sequence analysis was done for only 140 H1N1pdm09, H3N2 and B viruses directly from clinical samples. All samples had data on location and date of sampling
Wadegu (2016)(Wadegu et al., 2016)	Study nested in a sentinel surveillance network that collected consecutive samples during 4-year period from district hospitals in Kenya. No details on specific location and number of sentinel sites	Sequenced 92 viruses (21 H3N2, 18 seasonal H1N1 and 53 H1N1pdm09) archived samples (total not defined). All

		samples had data on location (country) and date of sampling.

Table S8: Circulating genetic clades among Africa H1N1 viruses sampled between 2001 and 2009

Clade	Year	Country	HA1 Amino Acid Substitutions	Study Reference	Representative strain
**H1N1 viruses**					
1	2001	Egypt	W252R	(Niang et al., 2012)	*A/New Caledonia/20/1999(H1N1)*
	2003	Senegal, Reunion			
	2004	Morocco			
	2005	Egypt, South Africa			
	2006	Egypt, Madagascar			
**2B**	2007	Egypt, Madagascar, Mauritius, Senegal	D35N, R159K, E274K	(Niang et al., 2012; Dia et al., 2013; Barr et al., 2010; Heraud et al., 2012; Wadegu et al., 2016)	*A/Brisbane/59/2007(H1N1)*
	2008	Algeria, Cameroon, South Africa, Ivory Coast, Madagascar, Ghana, Seychelles, Senegal, Kenya, Mauritius	D35N, R159K, E274K, A190T, D45N, K149R, R192K, E276K, D35N, E273K, E66K, S141N, G185A, A190T, K140E, K145R, N183S		
	2009	Kenya, Madagascar	K140E, K145R, N183S, D35N, E273K, S141N, G185A, E120D		
**2C**	2007	South Africa	T82K, Y94H, K141E	(Niang et al., 2012)	*A/St. Petersburg/10/2007(H1N1)*

Table S9: Circulating genetic clades among Africa H1N1pdm09 viruses between 2009 and 2018

Clade	Year	Country	HA1 Amino Acid Substitutions	Study Reference	Representative strain
**H1N1pdm09 viruses**					
**2**	2009	Kenya		(Gachara, 2014; Gachara et al., 2016)	***A/Czech Republic/32/2011(H1N1)***
	2010	South Africa	N31D, S162N, A186T, V272I	(El Rhaffouli et al., 2013), (Venter et al., 2012), (El Moussi et al., 2013a), (El Moussi et al., 2013b)	
**3**	2009	Cameroon		(Dia et al., 2013)	***A/Hong Kong/3934/2011(H1N1)***
	2010	Cameroon, Ghana, Senegal, Ivory Coast, Ethiopia	S183P, A134T	(Dia et al., 2013), (Gachara, 2014; Gachara et al., 2016), (El Moussi et al., 2013a), (El Moussi et al., 2013b)	
	2011	Tunisia	S183P, A134T, some with I96T or S74P	(El Rhaffouli et al., 2013), (El Moussi et al., 2013a), (El Moussi et al., 2013b), (Al Khatib et al., 2019)	
**4**	2011	Morocco	N125D	(El Rhaffouli et al., 2013)	***A/Christchurch/16/2010(H1N1)***
**5**	2010	Morocco	D97N, R205K, I216V, V249L	(El Rhaffouli et al., 2013)	***A/Astrakhan/1/2011(H1N1)***
	2011	Morocco, South Africa, Uganda, Tunisia	D97N, R205K, I216V, V249L, some with H138Q	(El Rhaffouli et al., 2013), (Klimov et al., 2012), (El Moussi et al., 2013a), (El Moussi et al., 2013b), (Soliman et al., 2020)	
	2017	Tunisia		(Al Khatib et al., 2019)	
**6**	2009	Kenya, Cape Verde	S203T, D97N, S185T	(Wadegu et al., 2016), (Owuor, 2021), (Dia et al., 2013)	***A/St Petersburg/27/2011(H1N1)***
	2010	Kenya, Morocco, Ghana, South Africa	S203T, D97N, S185T	(Wadegu et al., 2016), (Owuor, 2021), (El Rhaffouli et al., 2013), (El Moussi et al., 2013a), (El Moussi et al., 2013b),	
	2011	Kenya, Morocco, Ghana, South Africa, Tunisia	S203T, D97N, S185T, some with A186T and R205K or D222G	(Wadegu et al., 2016), (Owuor, 2021), (El Rhaffouli et al., 2013), (El Moussi et al., 2013a), (El Moussi et al., 2013b), (Klimov et al., 2012), (Mackenzie et al., 2019), (Barr et al., 2014)	
	2013	Tunisia		(Barr et al., 2014)	
**7**	2009	Egypt, Nigeria, Reunion, Mauritius, Tanzania, Kenya	S220T, D239E (in the complete HA protein)	(Dia et al., 2013), (Gachara, 2014; Gachara et al., 2016), (Owuor, 2021), (Pascalis et al., 2012)	**A/St.Petersburg/100/2011(H1N1)**
	2010	Senegal, Kenya, Mauritania, South Africa, Cameroon, Nigeria		(Dia et al., 2013), (Owuor, 2021), (Gachara, 2014)	
	2011	Kenya, Uganda		(Dia et al., 2013), (Owuor, 2021)	
	2012	Tanzania, Kenya, Madagascar	S185T, S143G, A197T	(Owuor, 2021), (Mackenzie et al., 2019), (Barr et al., 2014)	
6A	2014	Uganda	D97N, S185T	(Monamele et al., 2019)	***A/Hong Kong/5659/2012(H1N1)***
	2016				
6B	2013	South Africa, Ivory Coast, Nigeria	K163Q, A256T	(Monamele et al., 2019), (Tivane et al., 2018), (Sanou et al., 2018), (Kavunga-Membo et al., 2018), (Nkwembe et al., 2016)	***A/Norway/2417/2013(H1N1)***
	2014	Congo, Ethiopia, Egypt, Kenya, South Africa, Burkina Faso	K163Q, A256T	(Monamele et al., 2019), (Sanou et al., 2018), (Nkwembe et al., 2016), (Owuor, 2021)	
	2015	Congo, Kenya, Mozambique, Burkina Faso, South Africa, Tanzania, Ghana, Senegal, Mali, Madagascar, Zambia, Ethiopia, Nigeria,	K163Q, A256T, S69P, T120A, K208R, E235D, S84N, P271S	(Kavunga-Membo et al., 2018), (Mackenzie et al., 2019), (Owuor, 2021), (Tivane et al., 2018), (Sanou et al., 2018)	

		Central African Republic, Gambia, Morocco, Cameroon			
	2016	Kenya, Rwanda, Ghana	K163Q, A256T, S84N, P271S	(Mackenzie et al., 2019), (Owuor, 2021)	
6B.1	2015	Egypt, Ghana, Tanzania, Zambia, Nigeria, Ethiopia, Cameroon, South Africa, Congo, Egypt, Algeria, Kenya	K163Q, A256T, S84N, some with S162N and I216T	(Opanda et al., 2020), (Monamele et al., 2019), (Mackenzie et al., 2019), (Sanou et al., 2018), (Tivane et al., 2018), (Owuor, 2021), (Soliman et al., 2020)	***A/Michigan/45/2015(H1N1), A/Slovenia/2903/2015(H1N1)***
	2016	Ghana, South Africa, Tanzania, Ethiopia, Kenya, Cameroon, Niger, Madagascar, Ivory Coast, Mali, South Africa, Central African Republic, Senegal, Congo, Rwanda, Egypt, Kenya	K163Q, A256T, S84N, some with S162N and I216T	(Opanda et al., 2020), (Monamele et al., 2019), (Mackenzie et al., 2019), (Sanou et al., 2018), (Owuor, 2021), (Soliman et al., 2020)	
	2017	Ghana, Cameroon, Egypt, South Africa, Tunisia, Kenya	S162N, I216T, S74R, S164T, I295V, I150T	(Opanda et al., 2020), (Al Khatib et al., 2019)	
	2018	South Africa, Kenya, Egypt, Uganda, Madagascar	S162N, I216T, S74R, S164T, I295V, some with I150T or T120A	(Opanda et al., 2020)	
**6B.1a**	2018	Kenya		(Owuor, 2021)	***A/Paris/1447/2017(H1N1), A/Switzerland/2656/2017(H1N1)***
**6B.1a1**	2018	Kenya		(Owuor, 2021)	***A/Brisbane/02/2018(H1N1)***
**6B.2**	2014	Ethiopia, South Africa, Egypt	K163Q, A256T	(Monamele et al., 2019), (Soliman et al., 2020)	
	2015	South Africa, Cameroon, Ghana, Kenya, Burkina Faso, Mozambique, Madagascar, Central African Republic, Senegal, Mali	K163Q, A256T, E499K, some with S84N and R45K, V152T, V173I	(Opanda et al., 2020), (Monamele et al., 2019), (Sanou et al., 2018), (Mackenzie et al., 2019)	
	2016	Kenya, Mozambique, Cameroon	K163Q, A256T, E499K, some with S84N and R45K	(Opanda et al., 2020), (Monamele et al., 2019)	
**6B.3**	2013<br>2014	Egypt		(Soliman et al., 2020)	
**6C**	2012	Ivory Coast		(Sanou et al., 2018)	***A/Paris/2496/2013(H1N1)***
	2013	Togo, Madagascar, Ivory Coast, Ghana, Senegal, Kenya, Cameroon		(Sanou et al., 2018), (Owuor, 2021), (Soliman et al., 2020)	
	2014	Kenya, Burkina Faso, Ivory Coast, Senegal, Ghana, Cameroon	D97N, S185T, K283E, E499K, V234I, some with V30A, A186T, M257V, K142R, I286L	(Tivane et al., 2018), (Owuor, 2021), (Monamele et al., 2019), (Sanou et al., 2018), (Kavunga-Membo et al., 2018), (Nkwembe et al., 2016), (Mackenzie et al., 2019)	
**8**	2011	Ghana, Nigeria, Cameroon	A186T, V272A	(Klimov et al., 2012), (El Moussi et al., 2013a), (El Moussi et al., 2013b)	***A/Ghana/763/2011(H1N1)***
**9**	2012	Senegal	L32I, D86E, S128T, R259K, S263A, I460V, V520A	(Nkwembe et al., 2016), (Tivane et al., 2018)	
	2013	Ghana			
**Madrid**	2011	Tunisia	E172K, K308E, V47I	(El Moussi et al., 2013a), (El Moussi et al., 2013b)	***A/Madrid/SO8171/2010(H1N1)***

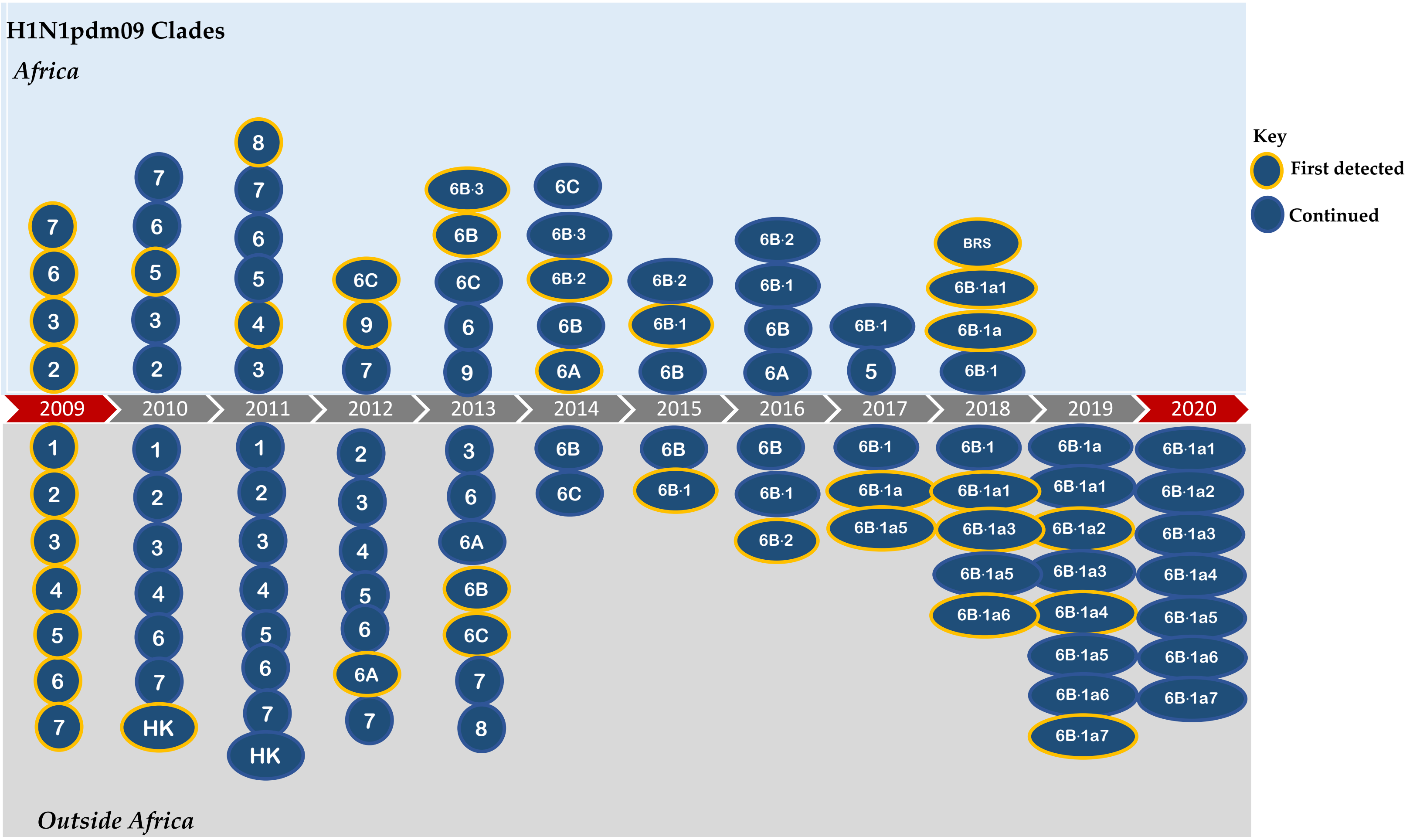

Characteristic substitutions in the HA1 protein of each clade H1N1pmd09 viruses: clade 1 (S128P), clade 2 (A186T, N31D and S162N), clade 3 (A134T and S183P), clade 4 (N125D), clade 5 (D97N, R205K, I216V and V249L), clade 6 (D97N), clade 7 (A197T, and many with S143G), and clade 8 (A186T and V272A). Abbreviations: The HK=A/Hong Kong/2212/2010(H1N1) clade has S128P, V199A, and I295V. All clades are based on the European Centre for Disease Prevention and Control (ECDC) influenza virus classification.

Figure S10. Viral diversification and distribution of genetic clades among H1N1pdm09 viruses that circulated in Africa versus elsewhere during the 2009-2020 seasons.

Table S11: Circulating genetic clades among Africa H3N2 viruses between 2004 and 2018

Clade*	Year	Country*	HA1 Amino Acid Substitutions	Study Reference	Representative strain
**H3N2 Viruses**					
**Fujian**	2003	Senegal, Madagascar, South Africa	Q156H	(Niang et al., 2012), (Besselaar et al., 2004), (Bulimo et al., 2008)	***A/Fujian/411/2002(H3N2)***
**Wellington**	2004	Senegal, South Africa	S159N	(Niang et al., 2012), (Bulimo et al., 2008)	***A/Wellington/1/2004(H3N2)***
**Brisbane**	2006	Ghana, Morocco	K140I	(Niang et al., 2012), (Bulimo et al., 2008), (Nyang'au et al., 2020), (Wadegu et al., 2016), (Byarugaba et al., 2011)	***A/Brisbane/10/2007(H3N2)***
	2007	Cameroon, Kenya, South Africa	K140I		
	2008	Kenya, Uganda	I140K, F193S, some with V112I and N144S, K173E K173Q		
	2009	Ghana, Egypt, Kenya, South Africa, Algeria, Madagascar, Tunisia, Ivory Coast, Nigeria, Cameroon	K173Q		
**Perth**	2008	Madagascar	N189K, E62K, N/S144K, K158N, I230V	(Heraud et al., 2012), (Byarugaba et al., 2011), (Niang et al., 2012), (Wadegu et al., 2016), (Nyang'au et al., 2020), (Al Khatib et al., 2019), (Klimov et al., 2012),	***A/Perth/16/2009(H3N2)***
	2009	Madagascar, Niger, Senegal, Uganda, Ghana, Egypt			
	2010	Niger, Kenya, Ghana			
	2011	Kenya			
**Victoria**	2009	Niger, Cameroon, Uganda, Kenya, Egypt, Ethiopia, Djibouti, South Africa, Nigeria, Algeria, Mauritius	K158N, N189K, T212A, N312S, some with S45N	(Heraud et al., 2012), (Nyang'au et al., 2020), (Byarugaba et al., 2011), (Owuor et al., 2020)	***A/Victoria/208/2009(H3N2)***
	2010	Niger, Ivory Coast, Cameroon, Madagascar, Kenya			
**3 (3A)**	2011	Ghana, South Africa, Nigeria	N144D, N145S, V223I	(Klimov et al., 2012), (Al Khatib et al., 2019), (Barr et al., 2014), (Soliman et al., 2020)	***A/Stockholm/18/2011(H3N2)***
	2012	Tunisia, South Africa, Ethiopia			
	2013	Tunisia			
**3B**	2011	Kenya, Tunisia, South Africa	N312S, A198S	(Owuor et al., 2020), (Nyang'au et al., 2020), (Barr et al., 2014)	***A/Athens/112/2012(H3N2)***
	2012	Tunisia, Egypt, Morocco, Madagascar			
**3C**	2011	South Africa	S45N, T48I, A198S, V223I, some with N312S, S145N	(Klimov et al., 2012) , (Nyang'au et al., 2020), (Barr et al., 2014)(Owuor et al., 2020)	***A/Victoria/361/2011(H3N2)***
	2012	Kenya, Senegal, Mauritius			
	2013	Mauritius			
	2014-2015	Kenya			
**3C.1**	2013	Tunisia, Ethiopia	Q33R, N145S, N278K	(Nyang'au et al., 2020)	***A/Texas/50/2012(H3N2), A/Berlin/93/2011(H3N2)***

**3C.2**	2013	Tunisia	N145S	(Al Khatib et al., 2019), (Soliman et al., 2020), (Monamele et al., 2017)	**A/Hong Kong/146/2013**
	2015	Egypt, Cameroon, Ivory Coast, Zambia, South Africa, Burkina Faso			
	2016	Egypt, Cameroon, Uganda, Congo, Tanzania, South Africa			
	2017	Egypt			
**3C.2a**	2014	South Africa, Kenya, Nigeria, Cameroon, Congo, Tanzania, Mauritius	L3I, K160T, N225D, F159Y, N128K, N144S, Q311H, N171K, M168V	(McAnerney et al., 2015), (Owuor et al., 2020), (Sanou et al., 2018), (Njifon et al., 2019), (Nkwembe et al., 2016)	***A/Hong Kong/5738/2014(H3N2), A/South Africa/R2665/2015(H3N2)***
	2015	South Africa, Kenya, Tanzania, Zambia, Senegal, Mozambique, Burkina Faso, Ivory Coast, Niger, Nigeria, Cameroon, Ghana, Morocco, Mauritius, Mali, Gambia, Central African Republic, Ethiopia, Tunisia	L3I, N144S, F159Y, N225D, Q311H, K160T, some with Q197R, N121K, R144K	(Tivane et al., 2018), (Mackenzie et al., 2019), (Sanou et al., 2018), (Owuor et al., 2020), (Njifon et al., 2019), (Owuor, 2021)	
	2016	South Africa, Ivory Coast, Cameroon, Madagascar, Kenya, Ghana, Algeria	L3I, N144S, F159Y, N225D, Q311H, K160T, S96N, P194L, Q197R, N121K, R144K, V529I, D53N, N128K	(Mackenzie et al., 2019), (Kleynhans et al., 2019), (Owuor et al., 2020), (Njifon et al., 2019), (Owuor, 2021)	
	2017	Kenya, Ethiopia	F159Y, N128K	(Njifon et al., 2019)	
**3C.2a1**	2016	South Africa	S96N, K160T, P194L, N171K, 1406V, G848E, N121K	(Kleynhans et al., 2019)	***A/Singapore/INFIMH-16-0019/2016(H3N2)***
		Ghana, Egypt, Mayotte, Algeria	F159Y, N171K	(Njifon et al., 2019)	
	2017	Ethiopia, Morocco, Tunisia, Congo, Tanzania, Kenya			
	2018	Egypt, Madagascar, Mauritius			
**3C.2a1b**	2016-2017	Kenya	L3I, K160T, N225D, N171K, K92R, H311Q	(Owuor et al., 2020), (Owuor, 2021)	***A/La Rioja/2202/2018(H3N2), A/South Australia/34/2019(H3N2)***
**3C.2a2**	2016	Kenya	L3I, K160T, N225D, R261Q	(Owuor et al., 2020), (Owuor, 2021)	
	2017	Zambia	T131K, R142K, R261Q	(Njifon et al., 2019)	***A/Switzerland/8060/2017(H3N2)***
	2018	Mauritius			
**3C.2a3**	2016-2017	Kenya	L3I, K160T, N225D, S144K	(Owuor et al., 2020), (Owuor, 2021)	***A/Norway/4849/2016(H3N2), A/Ghana/3294/2018(H3N2)***
**3C.2a4**	2017	Tunisia	N31S, D53N, N162K, I192T	(Njifon et al., 2019)	***A/England/74380294/2017(H3N2), A/Valladolid/182/2017(H3N2)***
**3C.3**	2012	Gambia, Ivory Coast, Algeria			***A/Samara/73/2013(H3N2)***

	2013	Nigeria, Ivory Coast, South Africa, Ghana, Kenya, Cameroon, Togo	Q33R, N145S, N278K, T120A, G78D, K140R	(Sanou et al., 2018), (Soliman et al., 2020), (Nyang'au et al., 2020), (Nkwembe et al., 2016), (Monamele et al., 2017), (Tivane et al., 2018)	
	2014	Nigeria, Burkina Faso, Zambia, Cameroon, Kenya, Congo, Senegal, Ethiopia, Egypt	T128A, R142G, V186G, some with S124N, G78D, I140R, Q173H		
	2015	Nigeria, Zambia, Mali, Kenya, Cameroon	T128A, R142G, S124N		
	2016	Ethiopia			
	2014	Burkina Faso, Ghana, Senegal, South Africa, Ethiopia, Tanzania, Madagascar, Cameroon, Nigeria	T128A, R142G, V186G, N225D, some with A138S, F159S, Y94H, K326R		***A/Switzerland/9715293/2013(H3N2)***
	2015	Cameroon, Nigeria	T128A, R142G, V186G, N225D, A138S, F159S, S198P		
**3C.3a**	2016	Uganda	T128A, A138S, F159S	(Sanou et al., 2018), (Nkwembe et al., 2016), (Mackenzie et al., 2019), (McAnerney et al., 2015), (Monamele et al., 2017), (Njifon et al., 2019)	
**3C.3b**	2014	Egypt	T128A, R142G, V186G, K83R, R261Q	(Nkwembe et al., 2016)	***A/Newcastle/22/2014(H3N2), A/Netherlands/525/2014(H3N2)***
**4**	2009	Egypt		(Soliman et al., 2020)	***A/Serbia/71/2011(H3N2)***
**5**	2011	Madagascar, Reunion	D53N, Y49H, E280A, I230V	(Klimov et al., 2012)	***A/Perth/10/2010(H3N2)***
	2010	Kenya			***A/Alabama/04/2011(H3N2), A/Norway/685/2011(H3N2), A/Johannesburg/107/2011(H3N2)***
	2011	South Africa, Tanzania, Kenya			
		South Africa			
**7**	2012	Kenya	S45N	(Owuor et al., 2020), (Klimov et al., 2012)	

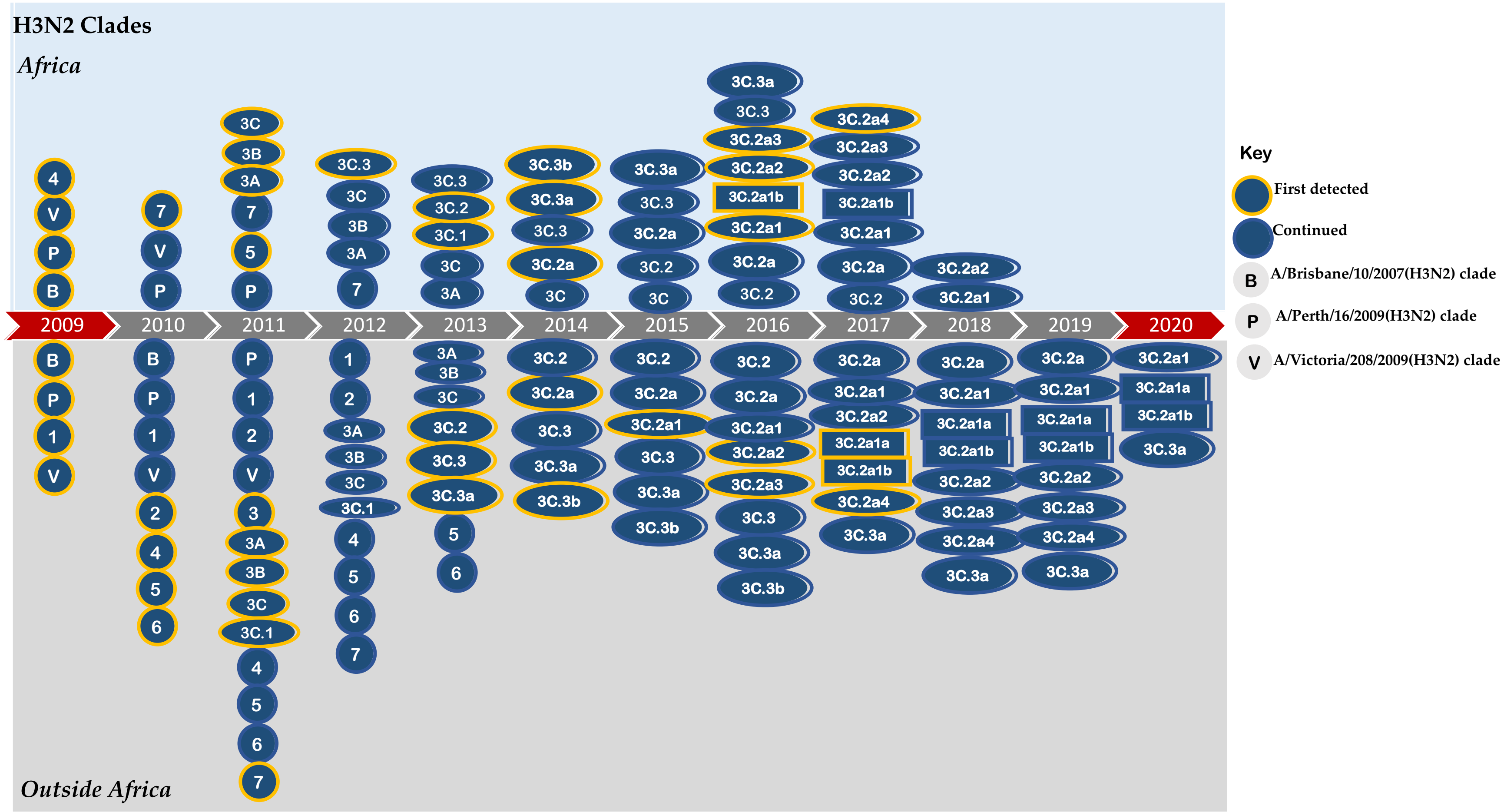

The main A/Perth/16/2009(H3N2) clade diverged into: clade 1 with substitutions P162S, I260M and R261Q, and clade 2 with substitutions N133D, R142G, T212A and V213A in their HA1 proteins. The main A/Victoria/208/2009 (H3N2) clade includes: clade 3 (V223I and some with N144D and N145S, some with A198S and N312S), clade 4 (T48A, K92R and N312S), clade 5 (D53N, Y94H, I230V and E280A), clade 6 (D53N, Y94H, I230V, E280A, and S199A), and clade 7 (N45S).

Figure S12. Viral diversification and distribution of genetic clades among H3N2 viruses that circulated in Africa versus elsewhere during the 2009-2020 seasons.
